# Existing evidence on the impacts of within-field farmland management practices on the flux of greenhouse gases from arable cropland in temperate regions: a systematic map

**DOI:** 10.1186/s13750-022-00275-x

**Published:** 2022-06-23

**Authors:** Alexandra Mary Collins, Neal Robert Haddaway, James Thomas, Nicola Peniston Randall, Jessica Jean Taylor, Albana Berberi, Jessica Lauren Reid, Christopher Raymond Andrews, Steven James Cooke

**Affiliations:** 1 Centre for Environmental Policy, Imperial College London, 109 Weeks Building, 16-18 Prince’s Gardens, South Kensington, London, SW7 1NE UK; 2 Leibniz-Centre for Agricultural Landscape Research (ZALF), Eberswalder Str. 84, 15374 Müncheberg, Germany; 3 Africa Centre for Evidence, University of Johannesburg, Johannesburg, South Africa; 4 EPPI-Centre, Social Science Research Unit, UCL Institute of Education, University College London, 18 Woburn Square, London, WC1H 0NR UK; 5 Centre for Evidence Based Agriculture, Harper Adams University, Newport, Shropshire TF10 8NB UK; 6 Canadian Centre for Evidence-Based Conservation, Carleton University, 1125 Colonel By Drive, Ottawa, ON K1S 5B6 Canada

**Keywords:** Carbon emissions, Global warming, Climate change, Climate mitigation, Arable farming, Farmland management, Evidence synthesis

## Abstract

**Background:**

Reducing the emissions of greenhouse gases (GHGs) is vital for mitigating climate change and meeting commitments to international agreements such as the COP 21 Paris Agreement of 2015. Agriculture is reported to account for approximately 11 percent of total global GHG emissions such that: the agricultural sector has an important role to play in meeting climate change mitigation objectives. However, there is currently little consensus on how farm management and interventions, along with interactions with in-field variability, such as soil type, affect the production and assimilation of GHGs in arable crop lands. Practical recommendations for farmers are often vague or generalised, and models (e.g. on the amount of nitrogen fertiliser applied) are used despite limited understanding of the influence of local conditions, such as the importance of soil type. Here, we report the findings of a systematic map of the evidence relating to the impact on GHG flux from the in-field management of arable land in temperate regions.

**Methods:**

We searched for, collated and catalogued research relating to the effects of in-field arable farming practices in temperate systems on GHG emissions. Results from 6 bibliographic databases, a web-based search engine and organisational websites were combined with evidence from stakeholders. Duplicates were removed and the results were then screened for relevance at title and abstract, and full-text levels according to a predefined set of eligibility criteria (following consistency checking). Relevant studies were then coded and their meta-data extracted and used to populate a systematic map database describing each study’s settings, methods and measured outcomes.

**Results:**

The mapping process identified 538 relevant studies from 351 articles. Nearly all of these (96%) were found from traditional research papers, with 42% from European countries and nearly half (203 studies) lasting for 12 months or less. Over half of all studies (55%) investigated multiple interventions with chemical fertiliser (n = 100), tillage (n = 70), and organic fertiliser (n = 30) the most frequently studied single intervention types. When combining individually studied and multiple interventions, the top three intervention types most frequently studied were: chemical fertiliser (n = 312); organic fertiliser (n = 176) and tillage (n = 158). Nitrous oxide was the most commonly studied outcome, with over double the number of studies compared to carbon dioxide, the next most studied outcome. Sandy loam and silty loam were the most commonly studied soils but there was a good distribution of studies across other types. However, studies predominately focused on humid sub-tropical (Cfa) and temperate oceanic (Cfb) climates, with hot summer Mediterranean (CSa) and warm summer Mediterranean (Csb) climate zones less represented.

**Conclusions:**

The mapping process identified clusters of research for chemical and organic fertiliser especially in relation to nitrous oxide emissions and for both carbon dioxide and nitrous dioxide in relation to tillage. Therefore, there is potential for further synthesis for these interventions. The spread of research across soil textures and in the humid sub-tropical and temperate oceanic climates may enable further synthesis to provide tailored in-field advice for farmers and provide an evidence base to inform subsidies policy. However, smaller amounts of research relating to biochar, cover crops, crop rotation, and nitrogen inhibitors highlight gaps where further research would be beneficial.

**Supplementary Information:**

The online version contains supplementary material available at 10.1186/s13750-022-00275-x.

## Background

Reducing the emissions of greenhouse gases (GHG) is vital for mitigating climate change and limiting global warming to the 1.5-degree aim outlined in the Paris Agreement. (http://unfccc.int/paris_agreement/items/9485.php). COP26, held in November 2021, confirmed commitment to this aim and outlined the urgent need to accelerate climate action [1]. It has been reported that agriculture accounts for approximately 11% of total global GHG emissions [2], the agricultural sector, including arable farming, thus has a vital role to play in meeting international and national climate change reduction objectives. This focus on the importance of agriculture is reflected in the Food and Agriculture Organisation (FAO) of the United Nations promoting the need for climate smart agriculture [3] and more regional examples such as the EU’s Farm to Fork Strategy for a fair, healthy and environmentally-friendly food system, which encourages soil management strategies to sequester carbon [4] and the US Government’s recent Climate Smart Agriculture and Forestry Strategy [5]. Targets are also set by national campaigning groups and governments. In the UK for example, the England and Wales National Union of Farmers’ have a goal of reaching net zero GHG emissions across the agriculture sector by 2040 [6], and targets have been set for the agricultural sector in The Scottish Government’s Climate Change Plan [7].

Atmospheric fluxes of GHGs, including carbon dioxide (CO_2_), methane (CH_4_) and nitrous oxide (N_2_O), are governed by the activity and turnover of soil microbial communities [8, 9]. The microbes are strongly regulated by changes in soil physical conditions, soil organic matter and nutrient availability that are themselves regulated by various aspects of agricultural management [10, 11]. For example, one of the driver of the production of N_2_O, which has a global warming potential 298 times larger than CO_2_ over a 100-year period [12], is the conversion of nitrogen (applied as fertilisers) to nitrate through processes such as nitrification and denitrification [9] and the release of N_2_O into the atmosphere. CH_4_ is only produced in anaerobic conditions by methanogenic bacteria but is also consumed by other methanotrophic bacteria when oxygen is available: therefore, acting as a sink for the CH_4_ produced in the soil [13]. CH_4_ production in agricultural fields is usually associated with high soil organic carbon, and often occurs within organic soils that are occasionally water-logged: these soils are considered as the main edaphic sources of CH_4_ from agriculture [14]. Agricultural soil management is therefore instrumental in defining the conditions for the soil bacteria and thus the production of GHGs from soils [15].

Despite the importance of soil management for GHG fluxes, there is little consensus at present regarding the effects of arable land management on the production and assimilation of GHGs and the mediating variables affecting the process. A wide diversity of land management practices is available to farmers (e.g. intensive tillage using mouldboard plough versus direct drilling or no till) but the effects of these different options on the emissions of GHG is poorly understood. As a result, practical advice for farmers is often overly general, and models (for example those based on the amount of nitrogen fertiliser applied) are used despite a poor understanding of the influence of local conditions, such as the importance of drainage, humus content and soil types. Whilst there have been specific studies investigating individual farming practices and GHG emissions, to the authors’ knowledge, there has been no broad collation and description of the evidence base related to in-field arable management and its impacts on GHG fluxes.

Here, we describe the conduct and findings of a systematic map of the experimental evidence concerning the impact of in-field farming practices on arable land in temperate regions on GHG flux.

### Stakeholder engagement

This systematic map was funded by the UK’s Natural Environment Research Council (NERC) under its *Environmental Evidence for the Future Programme* [16]. The topic was identified as a priority by the authors and discussed with stakeholders including; the UK’s Department of Environment Food and Rural Affairs (Defra), The Scottish Government, The Welsh Government, Environment Agency, Natural Resources Wales, The National Farmers Union (NFU), World Wildlife Fund (WWF), and the UK’s Natural Environment Research Council (NERC).

Stakeholders provided input to the protocol [17] by reviewing and refining the research question. Stakeholders were asked to identify potentially relevant literature, which was subject to the full screening process outlined below. Stakeholders were also asked to comment on the coding strategy so that information of greatest relevance to them has been included in the map.

### Objective of the map

Although the effects of in-field practices on GHG fluxes has been previously synthesised to some degree [18], there is no consensus yet regarding the influence of context (i.e. climate, soil texture, and organic matter content) on fluxes. Therefore, there is a need for a systematic map on the impact of arable farming practices on GHG emissions to collate and describe the evidence base that investigates these sources of heterogeneity across soils and climates. The need to inform land management and policy was identified, and the map developed with the support of (primarily UK-based) stakeholders. Therefore, the map has a focus on studies of relevance to the UK and temperate EU decision-making, however the map collates evidence across temperate climatic zones and so will also be of value for informing arable decision-making in temperate regions globally.

With this systematic map we have catalogued and described the evidence on the effects of arable, in-field farming practices on GHG fluxes, this includes climate and soil texture. Based on this review we have tentatively identified knowledge gaps and clusters that may warrant further research, via novel primary research and full systematic reviews, respectively.

The primary question for this systematic map was as follows:

*What evidence exists on the impacts of within-field farmland management practices on the flux of GHGs from arable cropland in temperate regions?*

This question can be broken down into the following key elements:

- *Population:* Arable farmland in temperate regions.
- *Intervention/exposure:* All within-field farmland management practices applied to arable cropland.
- *Comparator:* Without management, with different management, before management, with different intensities of management.
- *Outcome:* Fluxes of GHGs (CH_4_, N_2_O, CO_2_).
- *Study type:* Replicated observational and manipulative studies.

## Methods

This map followed detailed methods described in the a priori systematic map protocol [19] and was conducted according to the recommended systematic mapping methodology [20] and the guidelines provided by the Collaboration for Environmental Evidence (Collaboration for Environmental Evidence, 2018) and conforms to ROSES reporting standards [21] (see Additional file 5).

### Deviations from the protocol:

- We added five relevant reviews to the bibliographic checking process
- A public call for literature was not made
- A total of 5% and 4.4% consistency checks were conducted (at title and abstract, and at full text levels, respectively) instead of 10% for each, whilst this reduces the degree of consistency checking it was necessary because of the volume of search results and corresponds to 1,194 papers at title and abstract level and 180/375 at full text level. We do not believe this change affected our methods adversely, however, since thorough processes were applied throughout (see details on screening below) and a large number of papers were still used in consistency checking
- We extracted a smaller number of data items. We did not extract the following:
  o fertiliser quantity
  p presence of soil drainage
  q above-ground biomass
  r organic matter content

Reducing the data extracted in this way prioritised extraction of the rest of the data based on an initial analysis of the quality of the evidence base and how time consuming some of the items were to find and extract consistently. The creation and provision of the systematic map enables these details to be extracted at a later date if resources became available.

### Search for articles

#### Bibliographic databases and search strings

The following seven bibliographic databases were searched to find academic literature: AGRIS Agricultural database (FAO), Directory of Open Access Journals (DOAJ), PubMed, Scopus, EThOS, ProQuest Dissertations and Theses Global, and Web of Science Core Collections (institutional subscriptions that were used are detailed in Additional file 1). These databases were searched using the following English language Boolean search string adapted to each platform’s syntax appropriately. Here, we present the Web of Science format and provide each string as used in Additional file 1: Literature Searches:

TS = ((arable OR agricult* OR farm* OR crop* OR cultivat* OR field*) AND (plough* OR plow* OR till* OR "direct drill*" OR fertili* OR biosolid* OR "bio solid" OR organic OR manur* OR sewage OR compost* OR amendment* OR biochar* OR digestate* OR "crop residue*" OR "crop straw*" OR mulch* OR "crop rotat*" OR "break crop*" OR "grass ley" OR "clover ley" OR legume* OR "bioenergy crop*" OR "cover crop*" OR "grass clover" OR "cropping system*" OR "crop system" OR "winter crop*" OR "spring crop*" OR "summer fallow*" OR "catch crop*" OR intercrop* OR conservation) AND (CH4 OR methane OR CO2 OR "carbon dioxide" OR N2O OR "nitrous oxide" OR GHG* OR "greenhouse gas*" OR "green-house gas*") AND (flux* OR dynamic* OR emission* OR exchang* OR balanc*)).

Searches were conducted in English and performed using the subscriptions of Carleton University (see Additional file 1: Literature Searches for full details of the searches, settings, and dates of all bibliographic database searches).

#### Grey literature

Three broad types of grey literature searching were performed. Firstly, Google Scholar (previously demonstrated to be a useful source of grey literature [22]) was also searched using two simplified search strings (Additional file 1: Literature Searches). The first 250 results from each search string were exported into Excel and duplicates were removed. Results were screened for relevance and all relevant articles were included in the final article database.

Secondly, the websites of key organisations were searched using a combination of simplified search strings (e.g., arable AND greenhouse gas, agriculture and CO_2_; see Additional file 1: Literature Searches for full details) and manual hand-searching (i.e. systematic navigation through website publication pages). These websites were as follows:

- British Society for Soil Science (https://soils.org.uk/)
- Centre for Ecology and Hydrology (https://www.ceh.ac.uk/)
- Department of Agriculture, Environment and Rural Affairs, Northern Ireland (https://www.daera-ni.gov.uk)
- European Environment Agency (https://www.eea.europa.eu/)
- European Commission Joint Research Centre (https://ec.europa.eu/jrc/en)
- Environment Protection Agency Ireland (http://epa.ie/)
- Gov.UK (including Natural England) (https://www.gov.uk/search/)
- National Trust (https://www.nationaltrust.org.uk/)
- Natural Resources Wales (https://naturalresources.wales)
- Project Drawdown (https://www.drawdown.org/)
- Rothamsted Repository (https://repository.rothamsted.ac.uk/repository)
- Scottish Environment Protection Agency (https://www.sepa.org.uk/)
- Scottish Government (https://www.gov.scot/)
- SNIFFER (https://www.sniffer.org.uk)

#### Estimating comprehensiveness of the search

The main bibliographic database search string was tested for sensitivity by comparing results to a benchmark list of 25 articles known to be relevant to the review, suggested by the review team and the advisory group (see Additional file 2: Benchmark List). The minor adaptations in the search string resulting from this benchmarking that deviate from the string reported in the protocol [17] are reported in Additional file 1: Literature Searches.

#### Assembling a library of results

Articles retrieved from PubMed, Scopus, ProQuest Dissertations and Theses Global, and Web of Science Core Collections were exported and combined in EPPI-Reviewer [23] and duplicates were removed using EPPI-Reviewer’s automated and manual detection procedures. Due to restrictions in export capabilities, results from all other databases/resources were exported into individual Excel spreadsheets for duplicate removal and screening.

### Article screening and study eligibility criteria

#### Screening process

The final set of search results were screened using a two-stage approach, assessing title and abstracts and finally full text documents; including only those articles that were eligible in the subsequent stage. The number of articles excluded at each stage was documented and reasons for exclusion at full text were recorded (see Additional file 3). This is a deviation from the protocol in that detailed reasons for exclusion for all abstracts were not reported given the large volume of search results., although some categories for exclusion were captured (e.g., grassland not an eligible population).

Prior to screening at title and abstract, a consistency check was performed between two reviewers (JJT and CRA) on a random subset of 1194 from a total of 23,862 unique records (5%). This resulted in a Kappa statistic of 0.680 indicating a ‘good’ strength of agreement. All disagreements were discussed in detail, inclusion criteria definitions were clarified where needed, and screening was allowed to proceed.

Attempts were made to locate all articles that remained after title and abstract screening using the library subscriptions of Carleton University (see Additional file 1: Literature Searches) and the use of interlibrary loans. Prior to screening all articles at full text, a consistency check was again performed between the two reviewers (JJT and CRA) on a random subset of 180/351 full texts (51.2%). The resulting Kappa statistic was 0.68 indicating a ‘good’ strength of agreement and screening was allowed to proceed.

Additionally, the bibliographies of a random subset of five articles identified as relevant reviews during full text screening were hand searched for any relevant articles that were not captured in the searches described above. This step was not initially proposed in the protocol and was done as a means of confirming the comprehensiveness of the search. An additional two studies were included from relevant reviews.

At all stages of screening, reviewers did not make screening decisions on any articles of which they were authors.

#### Eligibility criteria

Articles were screened according to the criteria outlined in the protocol [19] that were developed through consultation with stakeholders. The eligibility criteria are outlined in Table 1.

**Table 1:** Eligibility criteria used in the systematic map

Key element	Inclusion	Exclusion
Eligible Subject	Arable farmland in temperate climates defined as fully humid and summer dry, i.e., Cfa, Cfb, Cfc, Csa, Csb, Cs in Köppen–Geiger classification (Kottek et al. [24]). Peatlands were included only when being used for agricultural purposes	Crops that are primarily found in tropical regions (e.g., rice, sugarcane, bananas) were excluded as these were considered as not relevant to the stakeholders. Grasslands, pastures, forests, and plantations were excluded, although grasses grown as bioenergy crops were included
Eligible intervention	Any farmland management practice applied to the crop or the soil, and that could be applied to entire fields. This included, for example: fertilisation; addition of amendments (e.g. lime); crop rotations; soil tillage	Practices such as buffer strips that are not feasible as whole-field interventions were excluded. Comparisons of different starting soil types/contents (e.g. phosphorus concentration, moisture content) were excluded if no actual intervention was present in the study. Land use change studies were excluded, i.e. where land is changed from arable to another type of use e.g. from arable to urban development
Eligible comparators	Different levels of a management practice or an absence of a particular practice, either spatially (nearby control fields or plots) or temporally (i.e. before a management practice was initiated)	Studies without a comparator at the intervention level were excluded. Studies that compared different crops (e.g., wheat vs corn) with the same intervention were excluded
Eligible outcomes	Fluxes of GHGs (CO_2_, CH_4_, N_2_O)	Precursors (e.g., HONO) were excluded
Eligible study designs	Any observational or manipulative experimental study	Modelling studies, greenhouse or laboratory studies and ex situ experiments were excluded
Eligible languages	Attempts to include articles in a range of languages in addition to English were made. Articles judged as eligible but in other languages are listed in additional information 3 but were not included in data extraction	

### Data coding strategy

Following full text article screening, we assembled an Excel database that describes all the relevant studies. If multiple studies were reported within one article, each study was entered as independent lines in the database. Here, we define a study to be an experiment that was undertaken over a specific time period at separate fields or experimental plots. Articles were identified as supplementary when the reported data could be found in a more comprehensive or complete source and assigned to the primary article instead.

Development of the map data extraction form and code-book through scoping activities and stakeholder discussions identified the following key variable and possible sources of heterogeneity for extraction:

- Bibliographic information
- Study location and details (e.g., geographic location, site identifier)
- Köppen-Geiger climate zone [24]
- Soil texture classification, land management history
- Study design and duration
- Intervention type
- Measured outcome (e.g., fluxes in CO2, N2O, CH4)
- Sampling methods (e.g., equipment type, equipment description, quantification method)

Coding options within these key variables were expanded upon throughout the process of scoping and extraction as different metrics were encountered (see Table 2 for the coding schema).

**Table 2:** Number of studies conducted in each eligible Köppen-Geiger climate zone

Köppen-Geiger climate zone	n
Cfb	236
Cfa	226
Csa	54
Csb	20
Not reported	2

‘n’ indicates the number of studies

To ensure that information was being extracted in a consistent and repeatable manner, two reviewers (JT and CRA) performed a consistency check on a subset of articles (5/351 included articles). Any disagreements were discussed and resolved, adding more detailed guidance to the extraction codebook when necessary. Coding and data extraction proceeded at the article level by a single reviewer (CRA), later replaced by JLR and AB (after the same consistency check as above was performed). Any queries were discussed with a third reviewer (JT). When a decision for a given query could not be made by JLR or AB, uncertainties were discussed and reconciled with the research team (JLR, AB, JT) in order to reach a consensus decision. When coding was complete, JLR and AB reviewed the database to ensure consistency in the use of codes across the three reviewers.

### Study validity assessment

Following standard guidance for systematic maps [20], formal critical appraisal of study validity was not conducted as part of this systematic map. We did however extract a selection of important metadata and coding variables that allows for a crude assessment of validity and for full critical appraisal in subsequent systematic reviews conducted on the map outputs. Relevant extracted meta-data include:

- Study design
- Duration of study
- Replication and randomisation

### Data mapping method and analysis

The number of papers found and retained at each stage of the review was collated and presented in a systematic map flow chart.

Descriptive information about the evidence covering the publication year and type, study country and climate zone, soil texture, study duration and design, spatial replication, interventions, and outcomes was collated and presented in a series of tables or figures.

Bubble plots and heat maps were created to provide a visual inspection of possible knowledge gaps and clusters, covering interventions against countries for the three measured outcomes, intervention type against climate zone and soil type.

## Results

### The systematic mapping process

**Fig. 1:**
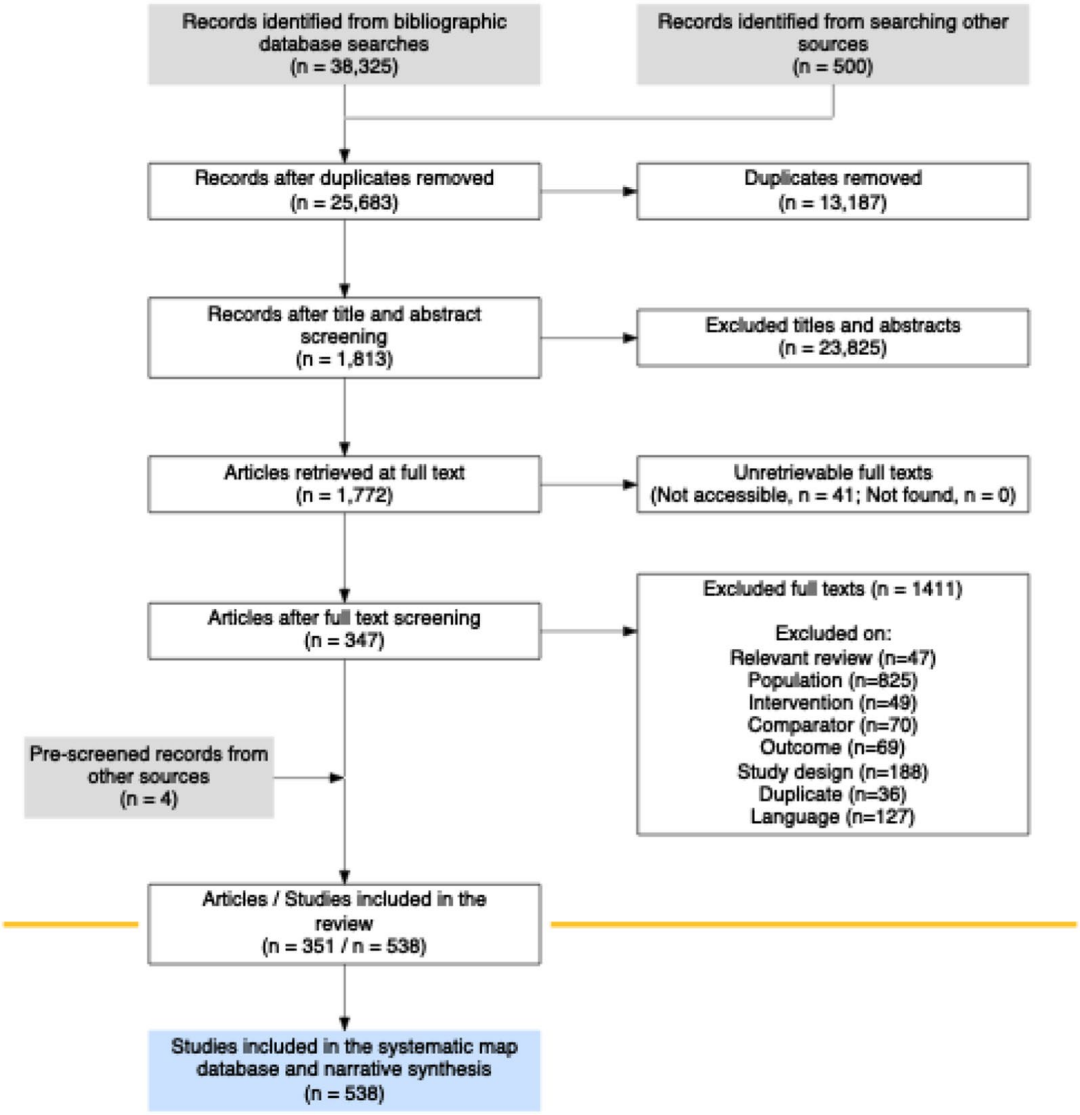
ROSES flow chart for the systematic map, showing the number of records retained at each stage of the review process. Produced using the R package ‘ROSES_flowchart’ [25]

Details of the number of records retained through each stage of the review process are provided in \* MERGEFORMAT Fig. 1. A total of 38,825 potentially relevant records were identified across all resources searched. A total of 25,683 unique records were screened for eligibility, with 347 eligible records following full text screening. Two additional studies were identified from websites and two from the relevant reviews. The final systematic map database contains 538 studies from 351 articles (Additional file 4).

### The map database and evidence portal

The systematic map database is provided in Additional file 4 and can be accessed via the online evidence portal (see below). The database can be searched, filtered and reused as necessary.

We have constructed an online evidence portal that summarises the project, the background, our methods, results and key messages. The evidence portal can be accessed via https://farming4climate.github.io/.

Key features of the web-based results are the interactivity and connectivity between the online versions of the static graphics provided herein. Namely, the individual features of each aspect of the plots (e.g. bars of the bar plots, bubbles in the heat maps) include ‘tooltips’ (popup boxes providing further information) and hyperlinks: the interactive ROSES flow diagram links to supplementary files and methodological detail, and the data visualisations link to filtered studies in the online version of the systematic map database. In addition, the map database and the evidence atlas include links to included articles’ corresponding author email addresses and links to full texts via Google Scholar Additional file 5.

### Descriptive information

#### Publication year

**Fig. 2:**
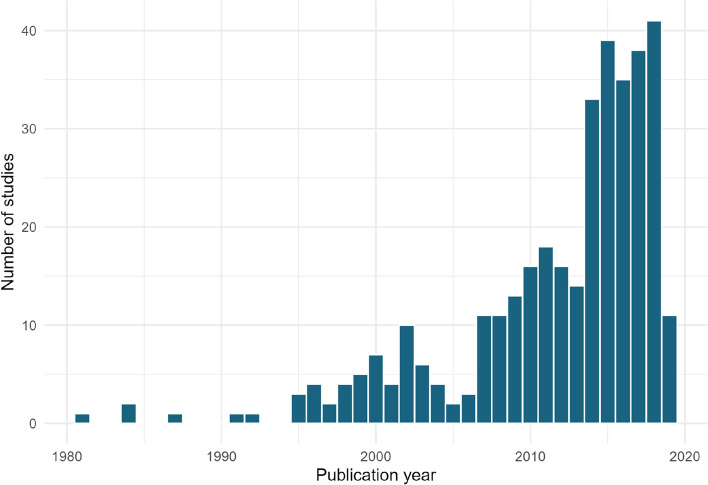
The final number of articles included in the systematic map by publication year

As expected, there has been a significant increase in the number of published articles on the topic over the last 20 years (Fig. 2). The earliest record in our database is from 1981. Since searches were performed in 2019, representation from this year is incomplete.

#### Publication type

Some 96% of articles in the map database are research papers, with only 8 theses, 7 conference papers, and 1 report. This may in some degree reflect the ease with which traditional research articles can be discovered, but may also be the result of the complex and expensive GHG measurement equipment needed for this type of research: it may be unlikely that unpublished reports would be conducted on a local or organisation scale.

#### Country

**Fig. 3:**
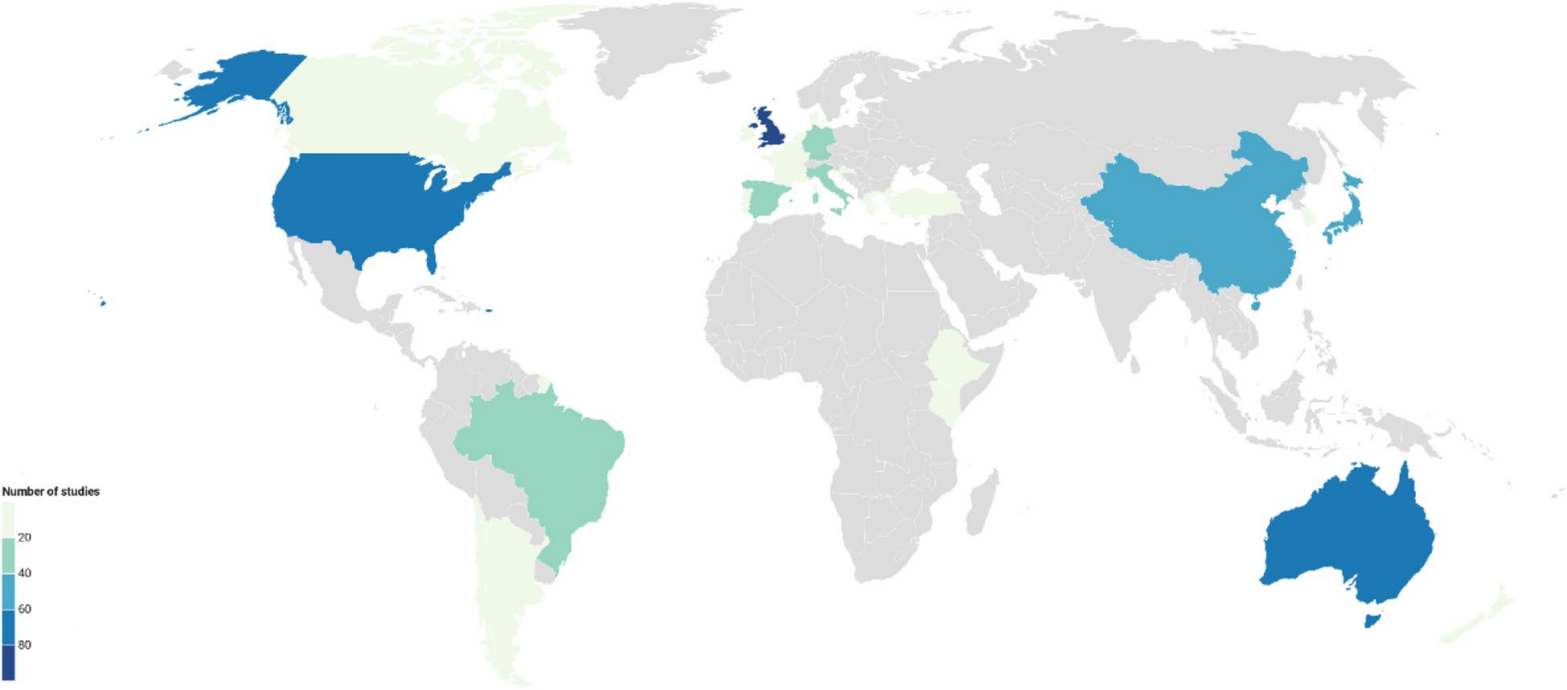
Choropleth showing the number of studies per country in the systematic map database

The choropleth map in Fig. 3 displays the number of studies per country. Some 3 countries each represented more than 10% of the total studies in the evidence base: United Kingdom (90), Australia (73), and USA (73). Nearly half of the evidence came from Europe (a total of 227 studies, 42% of all studies).

#### Climate zone

Table 2 displays the distribution of studies across climate zones. The most frequently studied climate zone was Cfb with 236 studies. Cfa was the second most studied zone with 226 studies. Two studies could not be located to a climate zone.

#### Soil texture

**Fig. 4:**
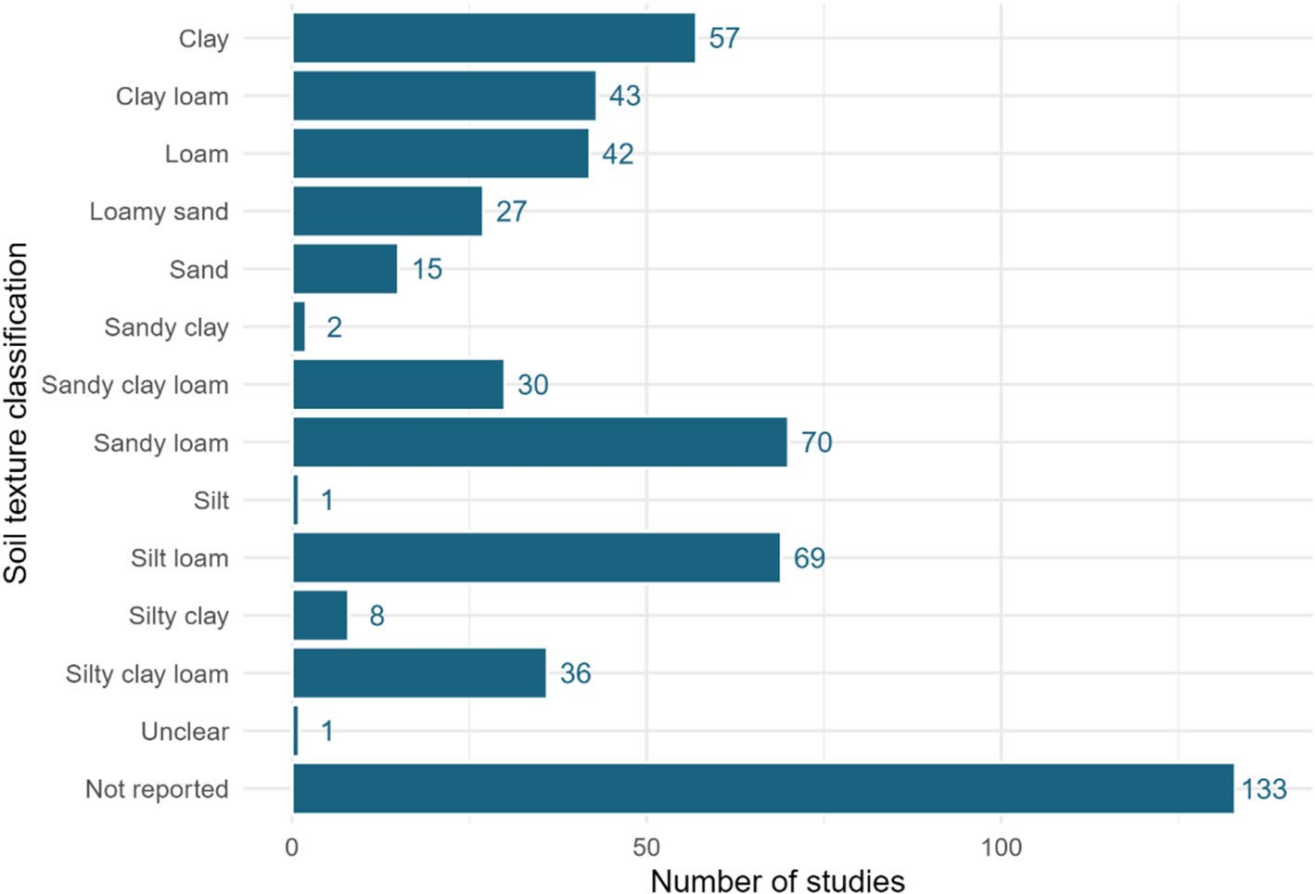
Soil texture classifications of studies in the systematic map

The most frequently reported soil texture information was from the USDA Natural Resources Conservation Service Soil Texture Classification System (a soil taxonomy system describing the components of sand, silt, clay and loam). Figure 4 shows the distribution of soil texture classifications using this system across the evidence base. Additionally, the systematic map (Additional file 4) provides free text comments regarding soil characteristics in column x.

A large number of studies (133 of 538) did not report the soil texture classification. Table 3 displays the soil texture data reported for studies not reporting this specific soil texture classification system, showing that 78 studies provided no data from any of the three soil classification systems, hampering synthesis of these data. Furthermore, there were few studies of the main soil types identified for temperate regions (Nortcliff, xx), e.g. Luvisols [5], Podzols [1], Cambisols [6] and zero for Fluvisols, Gleysols, Leptosols and Anthrosols.

**Table 3:** Soil classifications of studies not using the USDA Natural Resources Conservation Service soil texture classification system

USDA soil classification	FAO soil classification	n
Not reported		78
Andisol		14
	Andosols	13
	Cambisols	6
	Luvisols	5
	Acrisols	3
Vertisol		3
	Ferralsols	2
Oxisol		2
Alfisol		1
	Arenosols	1
	Hapli-Cutanic Luvisols (IUSS-WRB, 2007)	1
	Nitisols	1
	Podzols	1
	Vertisols	1
Ultisol		1

N = number of studies. Blanks indicate ‘not reported’

#### Field history description

Just over half of the studies in the systematic map (310 of 538) provided a description of the previous management practices used within the experimental fields.

#### Study duration

**Fig. 5:**
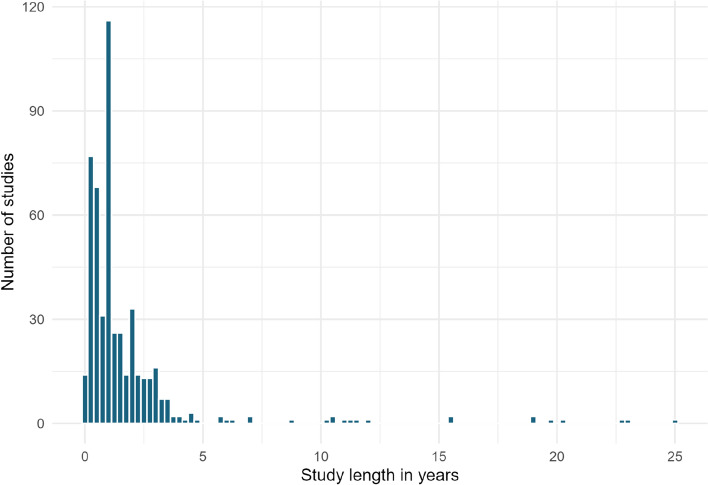
Study durations employed across the evidence base, collated into 3-month bins

The duration of investigation was reported in 511 of 538 studies. Figure 5 shows the range of study durations used across the included studies. Median study duration was 12 months.

**Fig. 6:**
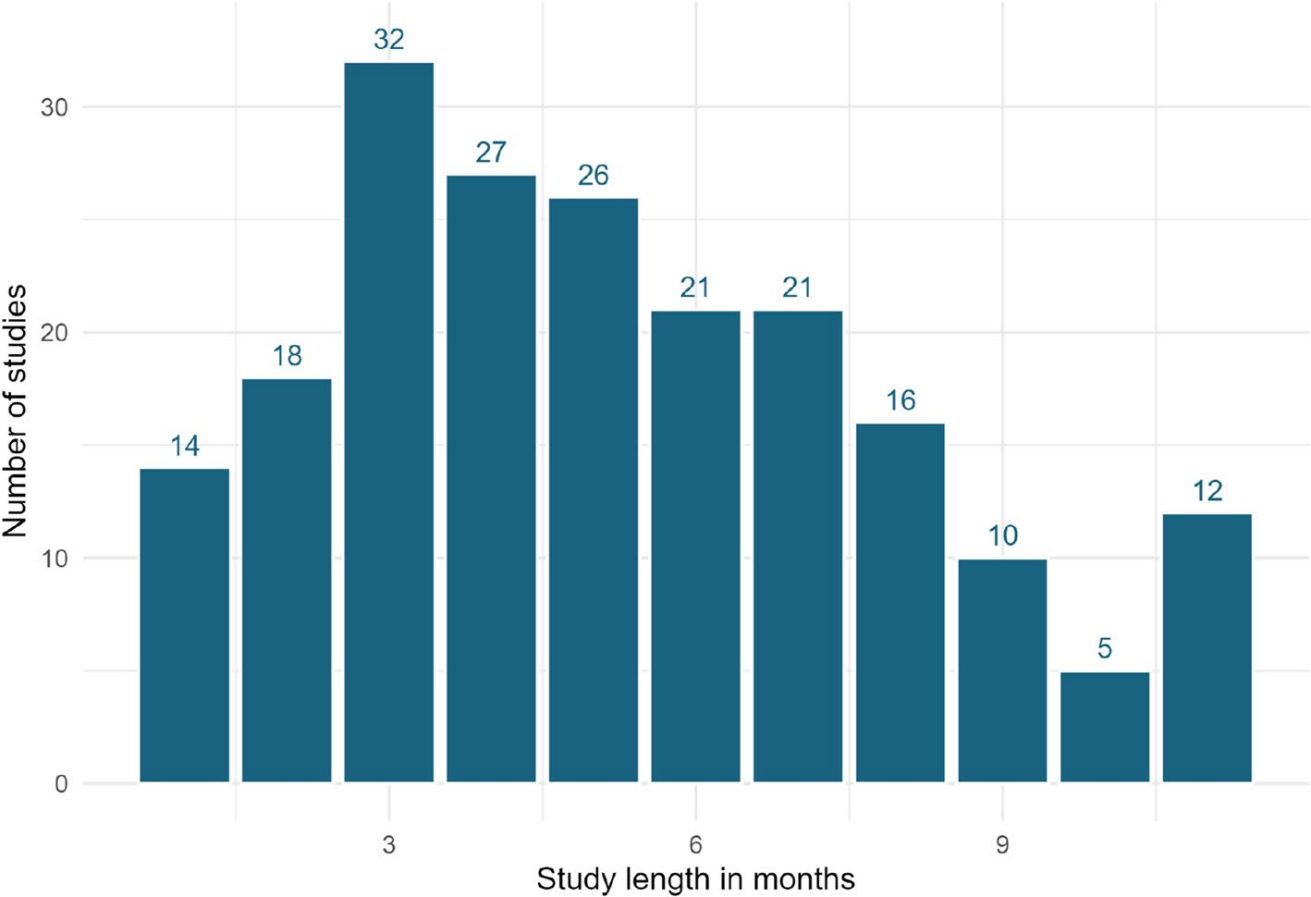
Study durations for investigations less than 1 year in length

For studies lasting less than one year (n = 203), the median study duration was five months (Fig. 6).

#### Study design

The most frequently employed study design across the evidence base was control-impacts (n = 534 studies). Before-after was much less common (n = 3). Study design was not reported in one study.

#### Experimental design

**Fig. 7:**
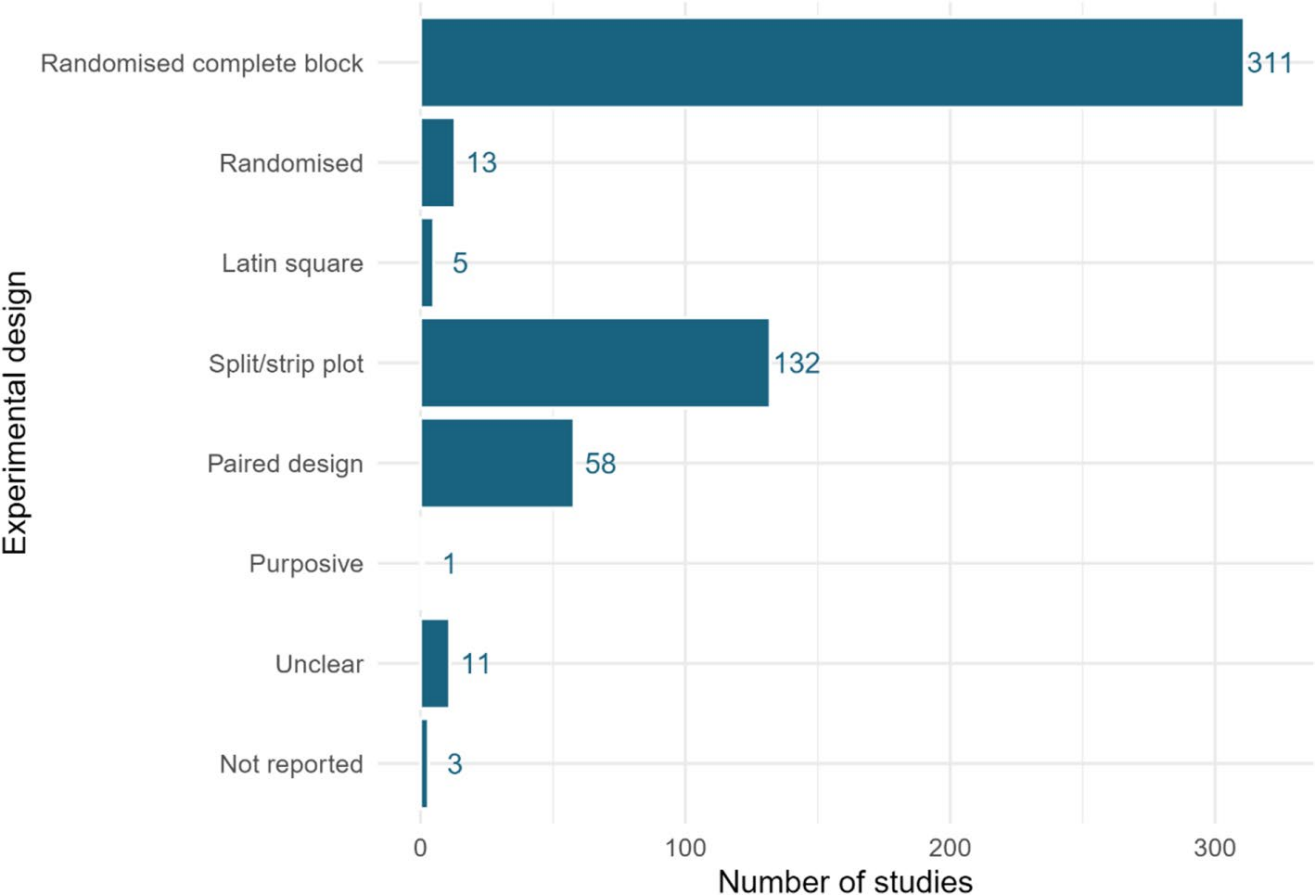
Experimental designs employed across studies in the systematic map

The most used experimental design in the included studies was ‘randomised complete block’, with 314 studies, with ‘split/strip plot’ the next most frequent (n = 132). Figure 7 displays the frequency of all experimental designs. The experimental design was not reported or unclear in 3 and 11 studies, respectively.

#### Spatial replication

**Fig. 8:**
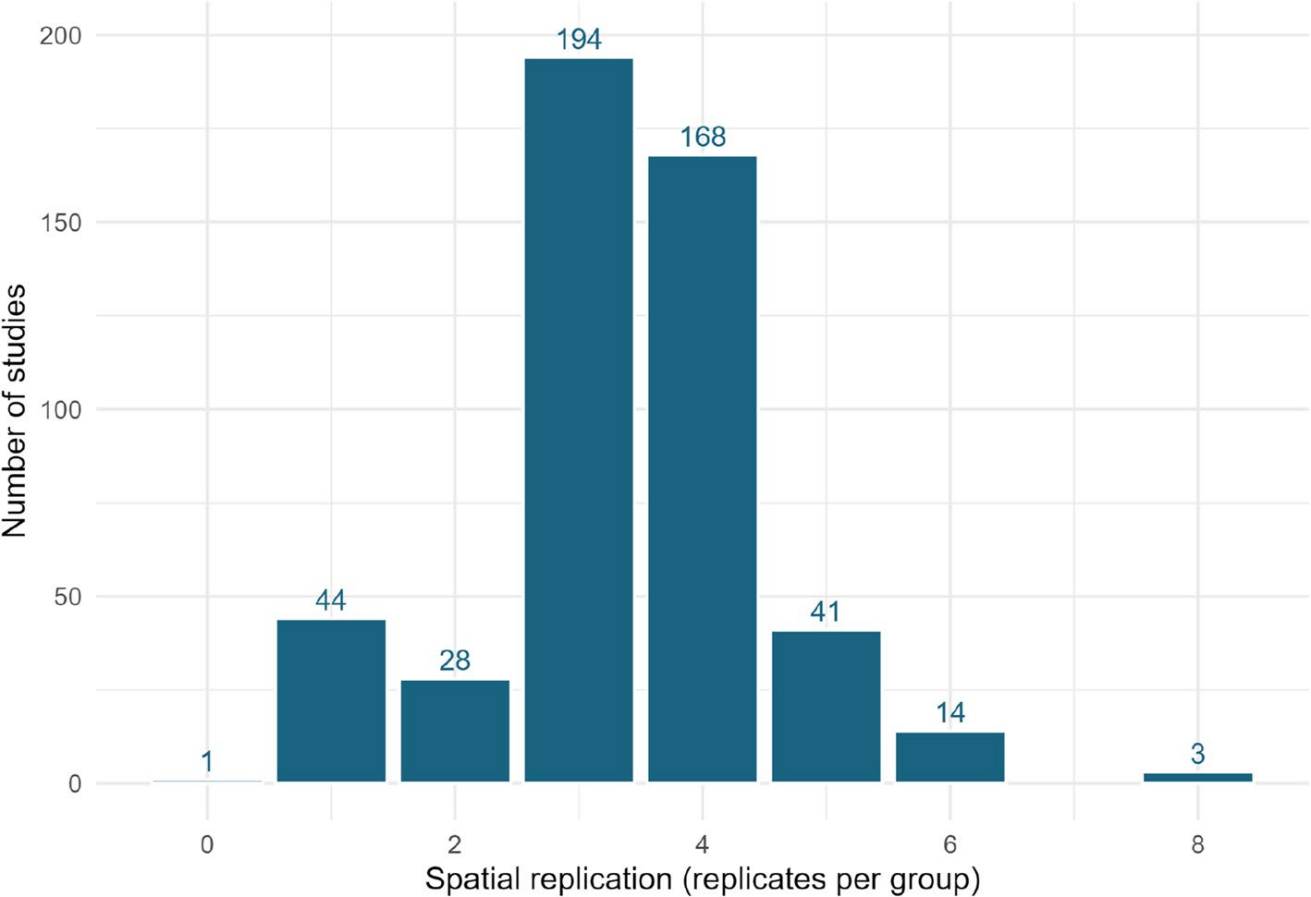
Study spatial replication (replicates per treatment group) across included studies in the map

Figure 8 shows the range of spatial replication across all studies. This demonstrates an overall very low level of true replication (no study used greater than eight spatial replicates). This is likely hindered by challenges in replicating field- or farm-scale experiments and the costs and time requirements associated with high levels of replication. The median level of true spatial replication was three replicates.

#### Temporal replication

Most studies in the map did not employ temporal replication (n = 510), with only 28 studies taking measurements for greater than 1 year before and 1 year after the intervention was applied.

#### Interventions

**Fig. 9:**
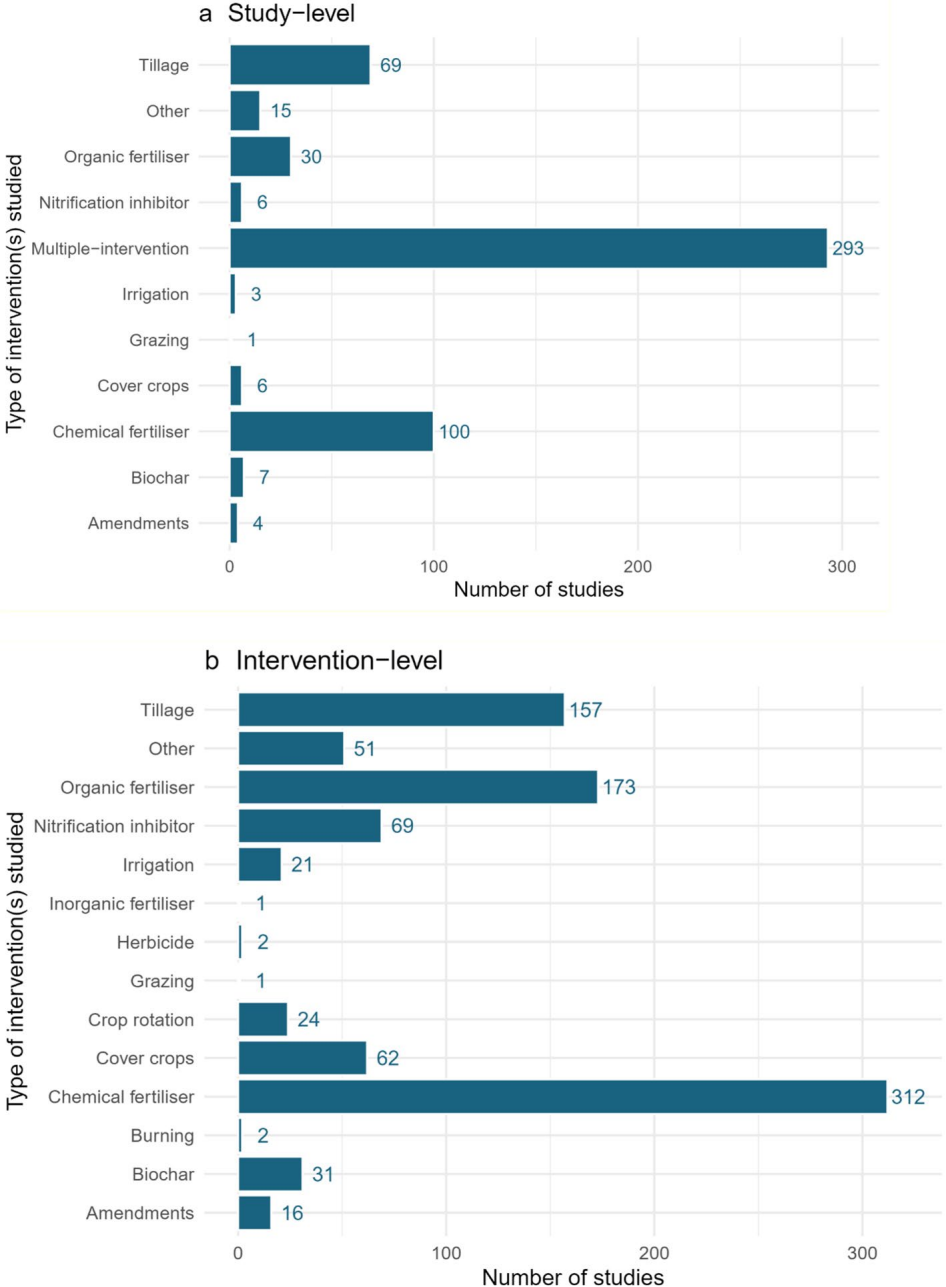
Types of interventions investigated in the evidence base, **a** at the study level, i.e. the broad category of management practice used, and **b** at the intervention level, i.e. the individual management practices investigated within each study

Figure 9 shows the types of interventions investigated in the evidence base. Here, we define intervention types as the broad category of management practice used, as opposed to treatments (see below), which are the individual management practices investigated within each study.

A total of 296 out of 538 studies (55%) examined multiple interventions together. The top three most frequently studied single intervention types were chemical fertiliser (n = 100), tillage (n = 70), and organic fertiliser (n = 30). Across all individual management practices, the top intervention types most frequently studied were: (1) chemical fertiliser (n = 312); (2) organic fertiliser (n = 176); (3) tillage (n = 158); (4) nitrification inhibitor (n = 72); and, (5) cover crops (n = 62).

#### Treatments

**Fig. 10:**
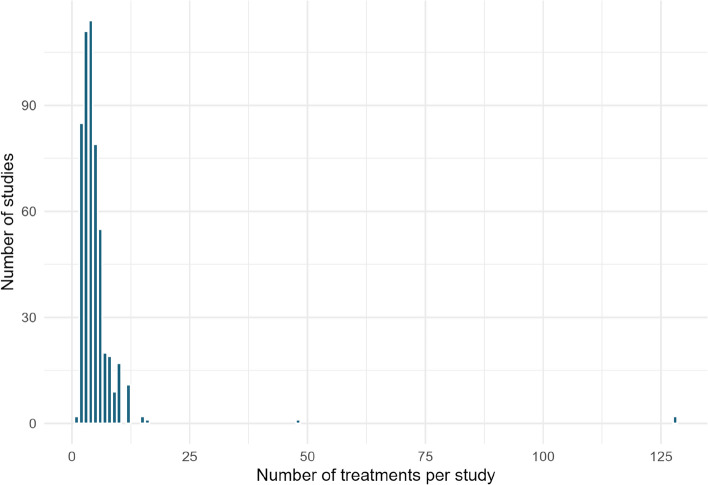
Number of treatments investigated in studies included in the systematic map

Within intervention types, studies often investigated multiple treatment levels/types (see Fig. 10). The median number of treatments was 4.

#### Outcomes

The most commonly studies outcome *across all studies in the map* was N_2_O (441), followed by CO_2_ (208) and CH_4_ (106). Table 4 shows where outcomes were measured together in combination. The most commonly studied combination was CO_2_ and N_2_O (58 studies), with all three outcomes (CH_4_ and CO_2_ and N_2_O) reported in 57 studies, and CH_4_ and N_2_O the next most commonly co-measured outcomes (n = 43).

**Table 4:** Outcomes measured together in studies measuring multiple outcomes in the systematic map

Outcomes	n
Carbon dioxide, Nitrous oxide	58
Methane, Carbon dioxide, Nitrous oxide	57
Methane, Nitrous oxide	44
Methane, Carbon dioxide	2

‘n’ indicates the number of studies

**Fig. 11:**
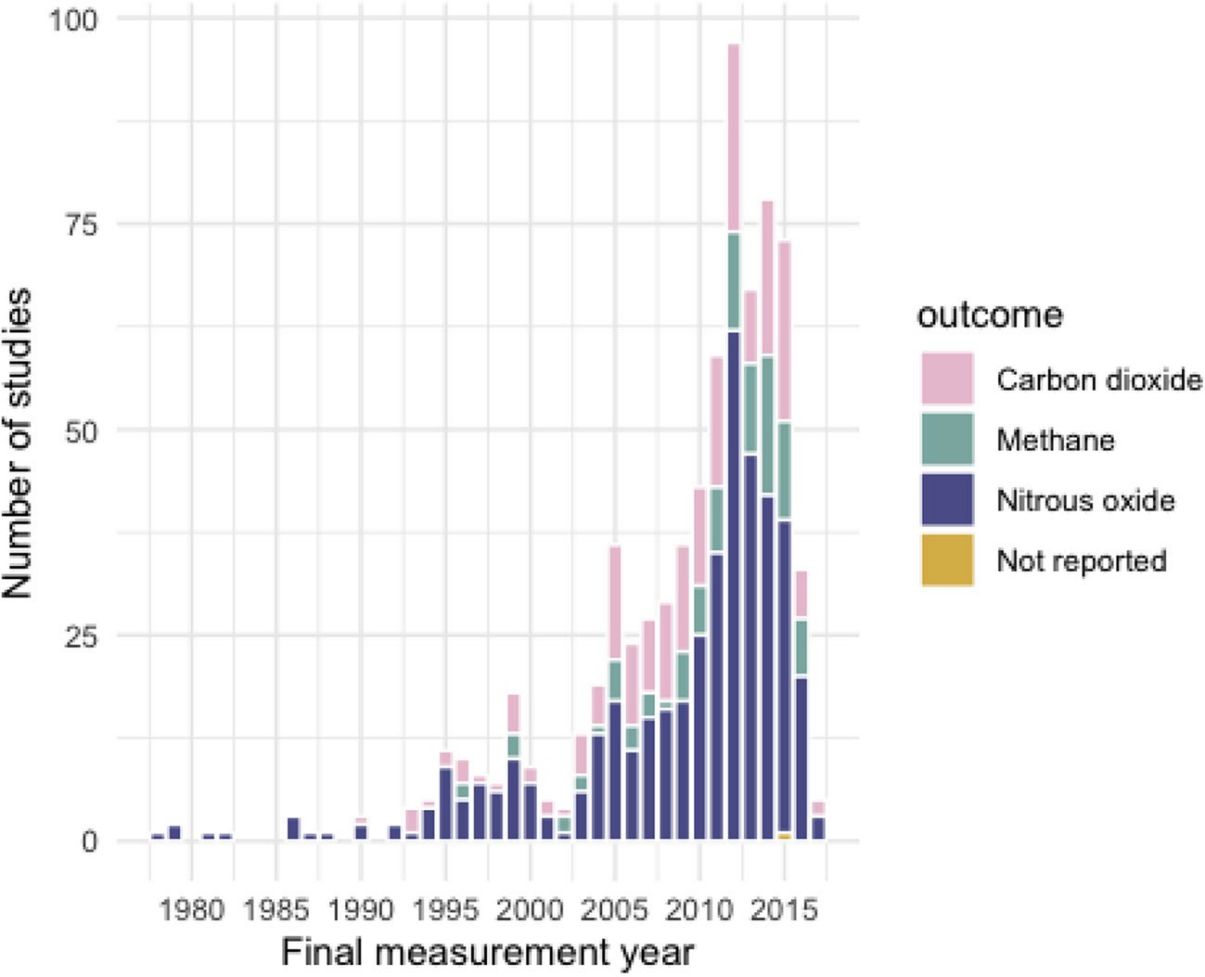
Measured outcomes across all studies by the final study measurement year

Figure 11 shows the distribution of measured outcomes across time (the final study measurement year), showing the consistent interest in nitrous oxide. These patterns give no indication of a change in attention to specific GHGs over time.

**Fig. 12:**
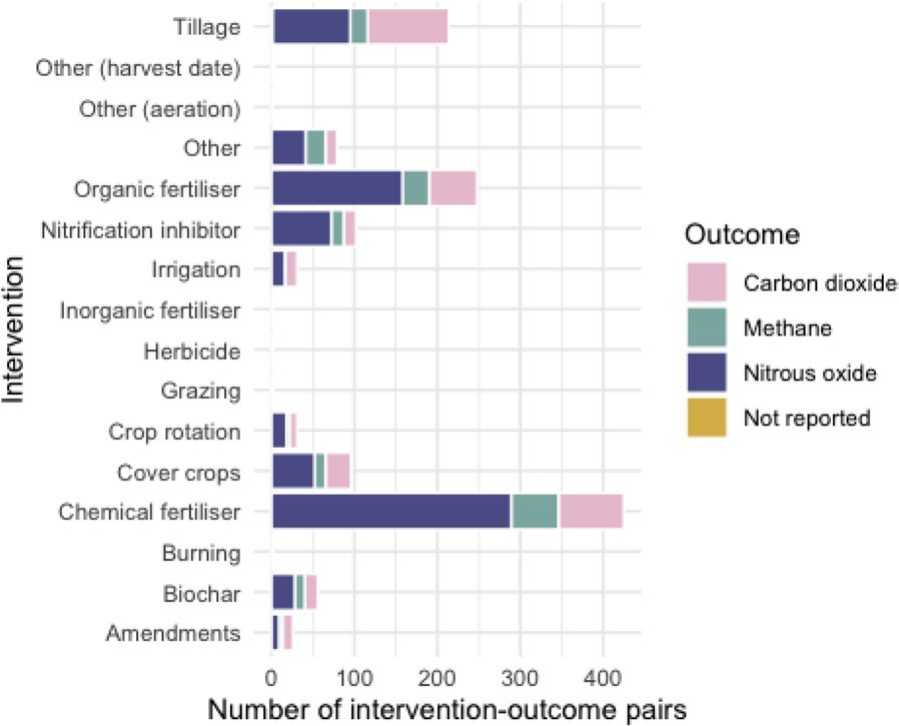
Measured outcomes across all interventions investigated

In Fig. 12, the total number of measured outcomes across all investigated interventions shows the prominence of nitrous oxide in research on fertilisers and nitrification inhibition. For tillage the split of outcomes investigated is more even across nitrous oxide and carbon dioxide.

**Fig. 13:**
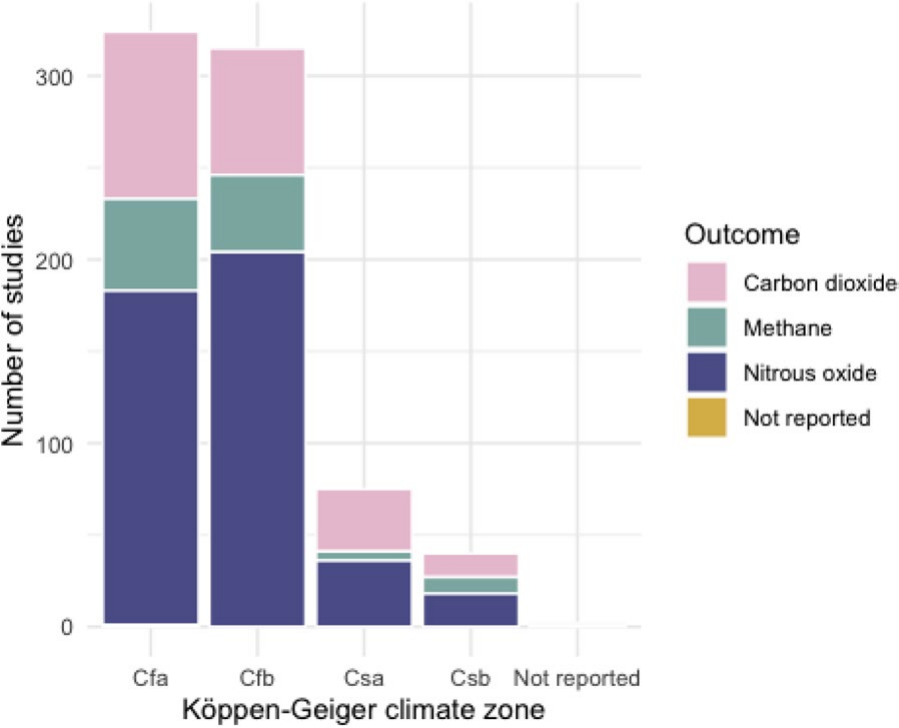
Measured outcomes across all studies by the climate zone

Fig. 13 shows the number of measured outcomes across all study climates. There is a somewhat smaller proportion of studies of nitrous oxide relative to other GHGs in Csa and Csb zones than Cfa and Cfb, but the total studies in this climate zone is also much smaller.

#### Outcome measurement methods

**Fig. 14:**
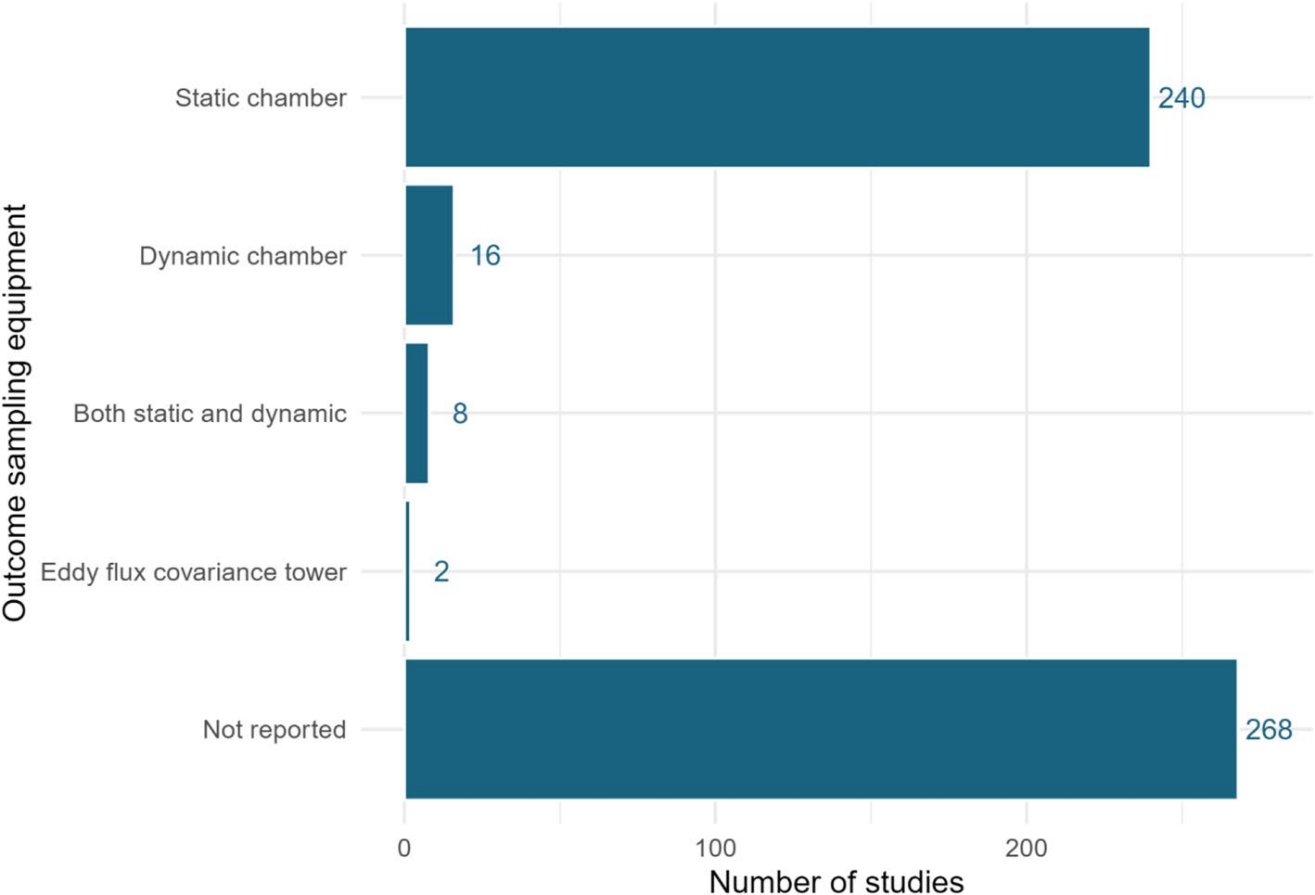
Experimental designs employed across studies in the systematic map

The most commonly reported measurement method was ‘static chamber’ (n = 243) (see Fig. 14). A substantial proportion of studies (n = 269) did not report the outcome sampling methods used. Whilst the percentage of studies not reporting outcome measurement method has reduced over time, i.e. 100% in the 1981 studies but just 24% of studies in 2018, there is still a relatively substantial proportion of studies not adequately reporting outcome method in contemporary studies.

Of the studies using chamber methods, open chambers were used in 17 studies, whilst closed were used in 231 (it was not possible to ascertain this information for the remaining studies). Opaque chambers were used in 141 studies, whilst transparent were used in 11 (it was not possible to ascertain this information for the remaining studies).

## Knowledge gaps and clusters

**Fig. 15:**
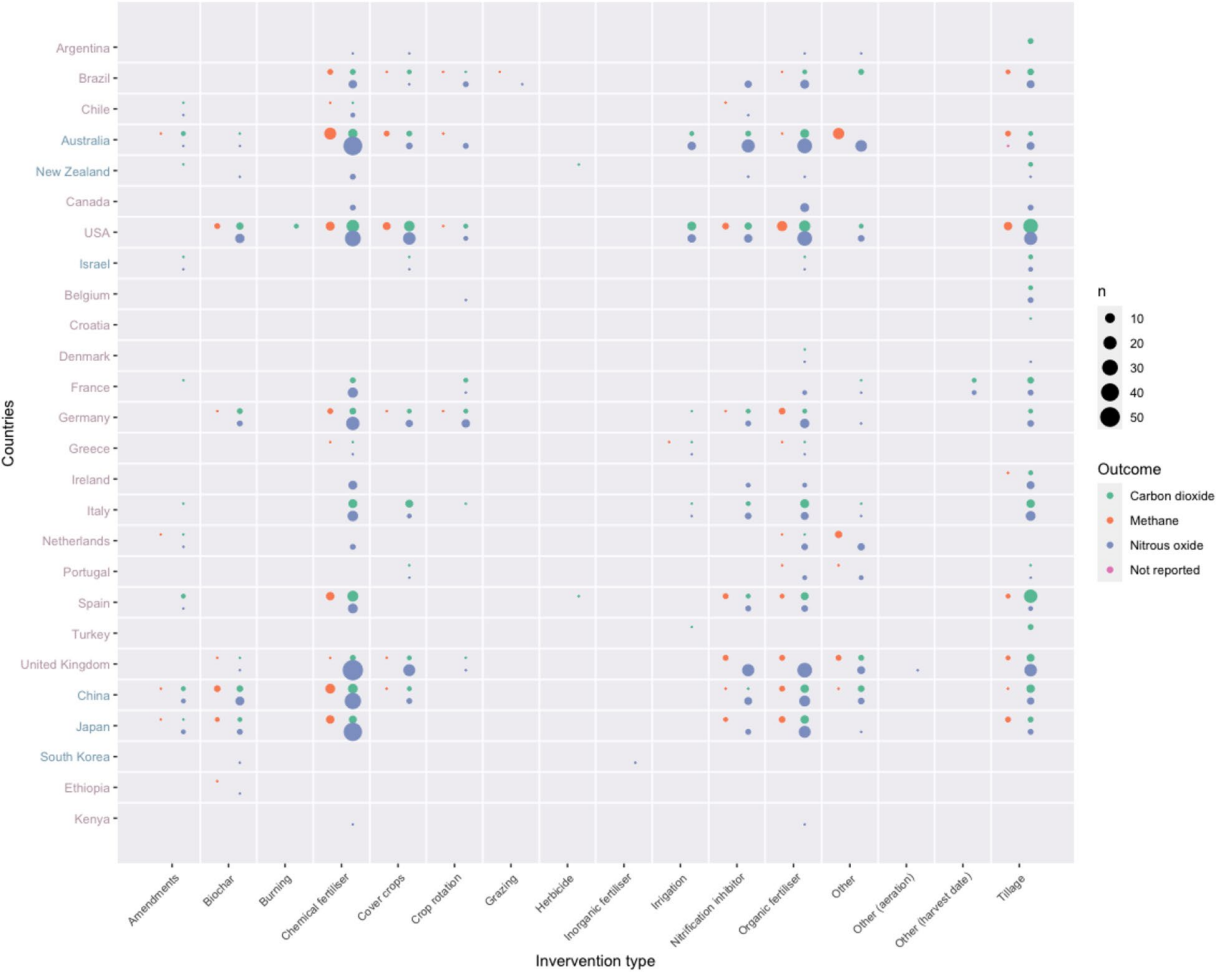
Bubble plot of intervention type against study country and measured outcome for studies in the systematic map. ‘n’ indicates the number of studies. Country labels in the y-axis are ordered and coloured by continent

In Fig. 15, interventions have been plotted against countries for the three measured outcomes. This highlights the dominance of research into chemical fertilisers, especially for nitrous oxide emissions across multiple countries, with higher amounts in Australia, USA, UK, China and Japan but also smaller amounts of research in Brazil, Chile, France, Germany, Italy and Spain. Whilst there is a smaller total volume of literature investigating organic fertilizers a similar pattern across countries can be seen, with dominance of research in Australia, USA, UK, China and Japan. Tillage is investigated across a wide range of countries, particularly for carbon dioxide and nitrous oxide, including countries where other interventions are not commonly investigated, including Israel, Belgium and Ireland. Interestingly research investigating biochar and crop rotation is mainly in countries outside of the EU, with the exception of Germany.

**Fig. 16:**
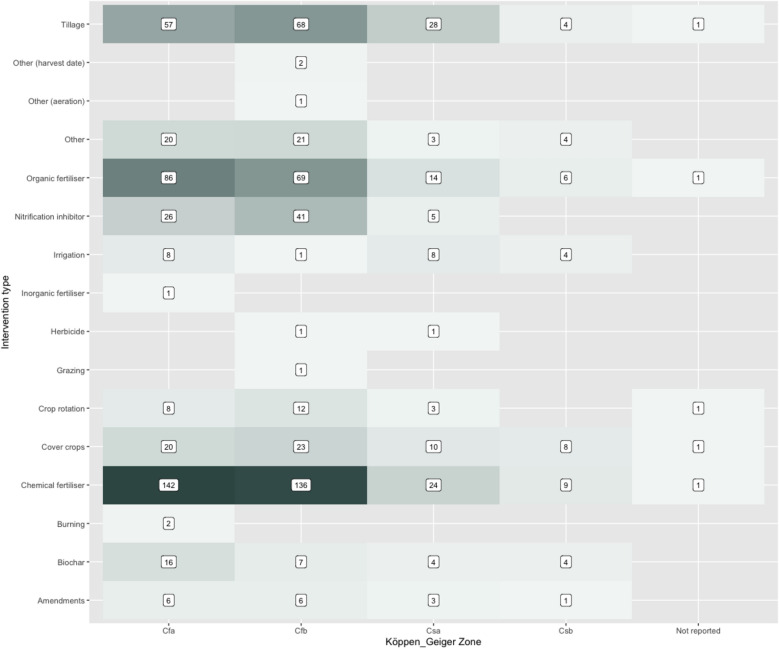
Heat map of intervention type against climate zone for studies in the systematic map. ‘n’ indicates the number of studies

In Fig. 16, interventions have been plotted against Köppen-Geiger climate zones in a heat map that shows the spread of evidence. This highlight the high amounts of research on chemical and organic fertilisers as well as tillage for Cfa and Cfb climatic zones, indicating potential for further synthesis and targeted advice for farmers regarding the use of these interventions in these climatic zones.

**Fig. 17:**
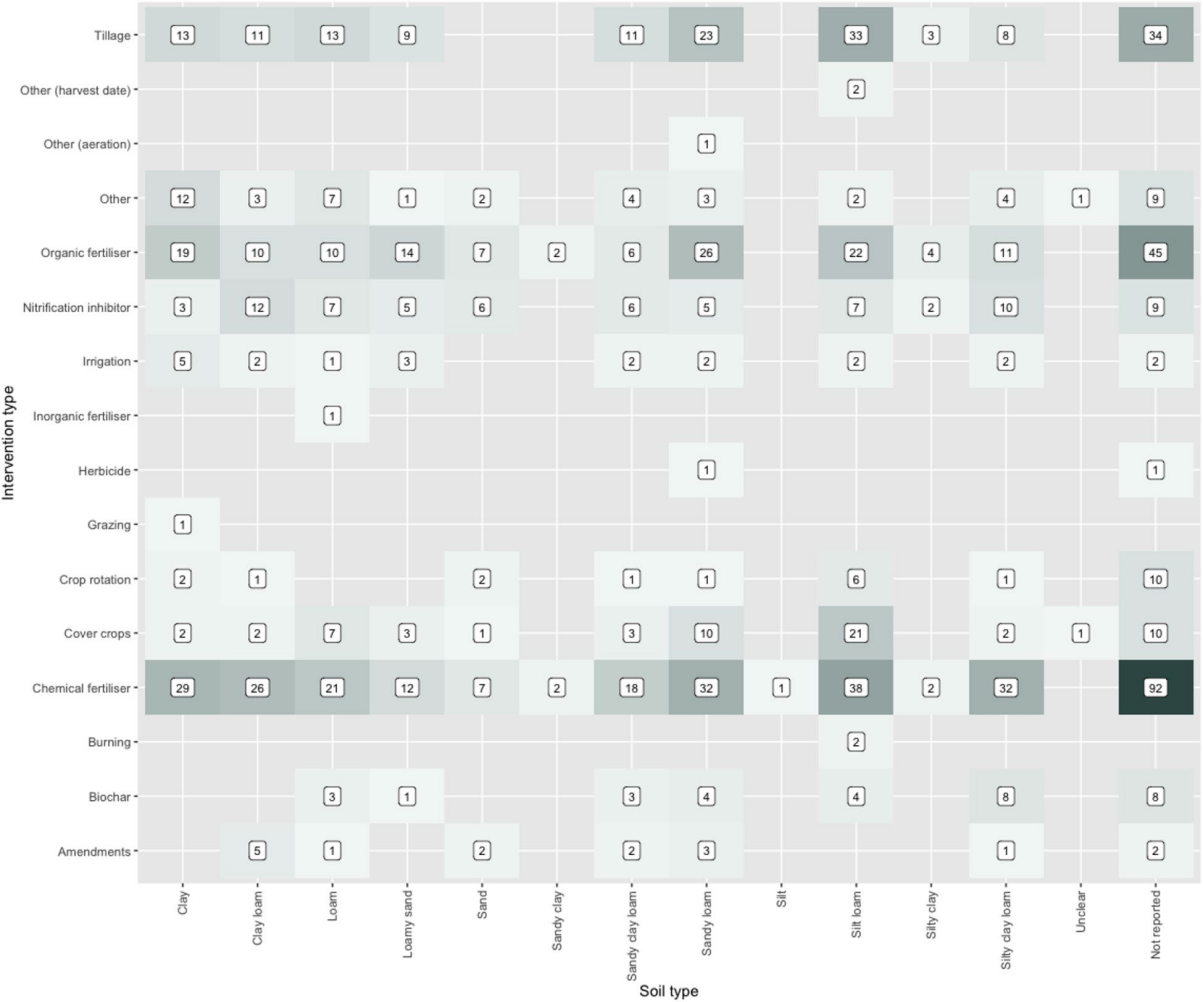
Heat map of intervention type against soil type for studies in the systematic map. ‘n’ indicates the number of studies

In Fig. 17, interventions have been plotted against soil type in a heat map that shows the spread of evidence. This shows a wide range of interventions investigated across sandy loam and silt loam soils, with a dominance of chemical and organic fertliser and tillage, as well as cover crops in silt loams. Although in smaller amounts there is a good range of interventions investigated across clay, clay loam, loam, loamy sand, sandy clay loam, and silty clay loams, indicating that there may be potential to provide targeted advice to farmers with differing soil conditions.

## Limitations of the map

### Limitations of the map due to the search strategy

Search strings only used English language, and studies were only included if the full text was reported in English. This may have influenced the distribution of studies found, over 40% of studies came from three English speaking countries. Additionally, whilst a range of grey literature searches were conducted these came from mainly UK websites, creating a potential bias in grey literature to UK studies. Only two studies were identified via website searches and so this is unlikely to have caused bias in the search results, since no geographical restrictions were applied to searches of bibliographic databases.

There are various factors that can influence soil emissions of greenhouse gases that were not recorded for this systematic map, e.g. soil pH and soil moisture [26], fertilizer quantity, soil matter content and drainage. Should any future synthesis be carried out, it may be important to consider these factors as reasons for heterogeneity between studies.

### Limitations of the evidence base

Over 90% of the literature was from peer reviewed publications. Should further syntheses be carried out, we would recommend testing for any bias in research findings linked to this potential publication bias in any evaluation of outcomes.

Nearly half (203 studies) lasted for 12 months or less, with very few studies lasting more than four years. These short-term studies do not take into account long-term variations in fluxes that may occur following or during mitigation due to external factors such as seasonality or climate. For example, both temperature and precipitation impact N_2_O emissions [27]. They also may not account for when an equilibrium may be reached or cumulative effects after a longer-term period. Studies lasting 12 months or less may be particularly vulnerable to missing longer term impacts of interventions as in most temperate regions this period only incorporates one growing season. Furthermore, most studies in the map did not employ temporal replication (n = 510), with only 28 studies taking measurements for greater than one year before and one year after the intervention was applied. Overall there was also very low level of true replication.

It is worth noting that nearly a quarter of studies (133 of 538) did not report soil texture. Soil texture is known to influence emissions of GHGs [26], e.g. percentage of clay in soils can affect emissions of N_2_O [28], although the relationship between texture and GHG appears to vary for each GHG, and also depends on other environmental factors.

The study designs should also be considered when interpreting results. Most studies had a control/intervention design, only 3 studies in total also incorporated a before and after treatment design. The number of spatial replications was also limited, with eight replicates being the maximum used.

Half of the studies (269 of 540) did not report the outcome measurement method used for testing, and most that did used static chambers. It is important that the measurement method be reported as each method, or chamber type where used, has limitations that may influence or restrict their GHG estimations [29].

## Conclusion

The number of studies regarding the flux of greenhouse gases from arable within-field management practices has been rising per year since the mid-1990s, which is likely to be as a result of increasing attention on climate change and mitigation measures. This rise has not been consistent however, with distinct jumps in 2007 and again in 2014. It is unclear why this is but could be as a result of changes in funding streams and areas of interest.

Of the 538 studies, 227 came from Europe (90 of which were from the UK), a further 73 each were from the USA and from Australia (373 of 538 from these regions). Since only two studies were found from grey literature searches, the focus on UK-based grey literature sources is unlikely to have had a substantial impact. The geographical distribution may reflect differences in policy and funding drivers or where there are clusters of academic and research expertise. The geographical distribution of studies also meant an uneven distribution in included climate zones, with the bulk of the studies from humid sub-tropical zones (Cfa), found in largely in parts of China, North and South America n = 226 and temperate zones (Cfb) found largely in North Western Europe n = 236. Hot summer Mediterranean (CSa) and warm summer Mediterranean (Csb) climate zones were also represented, with 54 and 20 studies respectively.

Most studies considered more than one intervention. This may be as a result of the costs involved with studying GHG emissions making it more cost effective to measure multiple interventions at once. Chemical fertiliser and organic fertiliser are interventions investigated the most at both the study and intervention level.

After chemical and organic fertiliser, tillage was the next most studied outcome. This focus on studies investigating the impact of tillage may reflect the need to resolve potential conflicts in the impact of tillage on GHG emissions, as researchers have reported both positive and negative factors relating to tillage depending on the GHG involved. For example, reduced tillage has been commonly reported to increase carbon sequestration but also to increased denitrification, which may lead to reductions in carbon dioxide but increase in nitrous oxide [30].

Nitrous oxide was the most commonly studied outcome, followed by carbon dioxide and then methane. Annual nitrous oxide studies in particular increased exponentially between 2002 and 2012 inclusive, with a notable jump in 2009/10. These increases could be linked to changes to research priorities due to increasing recognition of the importance of nitrous oxide as a greenhouse gas. For example, it was recognised as having a global warming potential 298 times that of carbon dioxide by the Intergovernmental Panel on Climate Change (IPCC) in their 2007 Assessment Report [31].

The key areas of study for nitrous oxide was in relation to chemical and organic fertilisers. This is probably unsurprising as fertilizer and manure application are often thought to have been the most important cause of direct emissions of nitrous oxide from agriculture during much of the period covered in this review [32]. For carbon dioxide, the pattern was slightly different, with tillage most studied, followed by chemical then organic fertilisers. Again, this may reflect the different impacts that tillage might have on carbon dioxide and nitrous oxide emissions.

### Implications for policy/management

There is increasing policy and practice interest in the role of agriculture in mitigating climate change and reducing carbon emissions. Climate–smart agriculture is an approach encouraged by the FAO [3] and included in strategies by various governments. In the US, for example, agriculture is a focus of a Government-wide approach to combatting climate issues, with the U.S. Secretary of Agriculture tasked to deliver recommendations for climate-smart agriculture [5]. Elsewhere, the European Parliament and Council’s recent agreement on the Common Agricultural Policy including higher environmental and climate ambitions than previously. Under this Member States must offer ecoschemes which will reward farmers for implementing climate and environmentally-friendly practices, allocating 25% of income support to these schemes. Additionally, 35% of rural development funds will be allocated to agri-environment commitments including climate beneficial practices [33]. Proposals under a new EU carbon farming initiative [34] and soil strategy [35], focus even further on the role of carbon reductions in agriculture. Within England, the Government has announced plans for three Environmental Management Schemes to reward farmers and land managers for delivering environmentally sustainable actions, with reduction of and adaptation to climate change listed as one of the key goals of the schemes [36].

In order for these schemes to support climate mitigation the practices they support needs to be evidence based. This systematic map highlighted research clusters for chemical and organic fertilizer management as well as tillage, indicating that further synthesis in these areas is likely to be possible and to support evidence informed practice for these interventions. Research across a range of soil types and particularly for sandy loam and silt loam soil types may allow tailored advice to farmers on these soil types, e.g. providing recommendations to farmers based on the in-field conditions. This may also be the case for farmers in Cfa and Cfb climates due to clusters of research identified here. This systematic map provides a database of studies that could enable further analysis in the clusters identified. This is particularly valuable to those seeking to support arable farmers in climate it would enable recommendations on management practices and interventions to support evidence informed climate mitigation advice, along with the design of incentive schemes. In parallel to these syntheses further research in how best to communicate the results and enable knowledge exchange with arable land managers and policy and practice decision makers would also be of value.

The large number of studies investigating chemical fertilisers (312 studies) may reflect that emissions from synthetic fertilisers are a key policy concern, with scientists from the IPCC predicting that current global growth rates will lead to this becoming the second largest agricultural source of GHG emissions in the next 10 years [37]. There are signs that in Europe, policies and agreements, along with changes in cropping patterns and the impact of increased fertilizer prices, may have limited this growth however, as emissions from chemical fertilisers in Europe decreased since 2010. The majority of recent global emissions came from developing countries (70%) [37]. Many of these regions would have fallen out of the scope of this review but may be where future policy needs to focus.

The smaller amounts of research investigating biochar, cover crops, crop rotation and nitrogen inhibitors may prevent evidence-based policies and practice advice for farmers. The small amounts of research found on biochar and crop rotation within EU countries is surprising, especially for crop rotation as crop diversification is now a requirement for greening payments to farmers, which make up 30% of EU countries financial support to farmers [38]. Whilst biochar is recognised for its ability to improve soil quality and sequester carbon [39], the low levels of research found investigating it as an intervention may be as a result of it being classified as a waste product of the energy industry and therefore subjected to the European Directive on Waste [40], which hinders its possible agricultural use [41] as well as uncertainties regarding its cost benefit [39].

### Implication for research

As noted above this systematic map has identified gaps relating to the investigation of biochar, cover crops, crop rotation and nitrogen inhibitors on the flux of GHG. Research addressing these gaps would enable the potential of these interventions for mitigating climate change to be assessed and evidence-based policies and practices implemented.

To improve the evidence base, future studies should aim to use experimental designs that incorporate before/after control intervention design, with temporal and (where relevant) spatial replicates. There is also a need to consider more long-term studies (studies that cover more than 12 months). Reporting could also be improved in many studies. For example, researchers should aim to report soil texture and other soil characteristics, such as soil mineral density and organic matter content, as this was not always done.

### Implications for synthesis

Chemical fertiliser, organic fertiliser and tillage were the most studied interventions and offer areas for further synthesis. For example, specific fertiliser type e.g. urea, ammonium nitrate, manure etc., could be further investigated, along with potentially different application times, amounts and methods, so as to inform management practices. Synthesis should investigate interactions with soil types, pH and soil moisture as well as across climatic zones so that targeted advice at the farm and field level can be made.

Further synthesis regarding reduced tillage would also be particularly valuable as tillage has been commonly reported to increase carbon sequestration but also to increased denitrification, which may lead to reductions in carbon dioxide but increase in nitrous oxide [30]. Insights from this synthesis could help to clarify the role of reduced tillage in climate mitigation and provide policy and practice advice.

Given the increasing volume of literature identified by this map and the significance of it to climate mitigation and policy objectives, regular updates to the map to include new research are recommended. Exploring the use of computer assistance in this so that maps are continually updated to include relevant research as and when it is made available [42], could be particularly valuable.

## Supplementary Information

**Additional file 1.** Literature Searches.

**Additional file 2.** Benchmark studies.

**Additional file 3.** Excluded studies.

**Additional file 4.** Systematic map.

**Additional file 5.** ROSES form.

## Data Availability

All data are made available in additional files or linked to externally on long-term data repositories.
