# Supplementary material for: Existing evidence on the impacts of within-field farmland management practices on the flux of greenhouse gases from arable cropland in temperate regions: a systematic map
Source: Environ Evid. 2022 Jun 23;11:24. doi: 10.1186/s13750-022-00275-x (PMC11378830; doi:10.1186/s13750-022-00275-x)
Supplement: Supplementary file 1 — Additional file 1. Literature Searches. [file 13750_2022_275_MOESM1_ESM.docx]

# **What are the impacts of within-field farmland management practices on the flux of greenhouse gases from arable cropland in temperate regions? A systematic map protocol**

Seven bibliographic databases will be searched to find academic literature, including: AGRIS Agricultural database (FAO), Directory of Open Access Journals, PubMed, Scopus, EThOS, ProQuest Dissertations and Theses Global, and Web of Science Core Collections.

### Web of Science – Core Collection

Note: “Topic” search in Web of Science includes: title, abstract, keywords, keywords plus

Table 1. Metadata from Web of Science – Core Collection Search Strategy

| Search string | Restrictions | Returns  [Date] |
| --- | --- | --- |
| TS=((arable OR agricult* OR farm* OR crop* OR cultivat* OR field*) AND (plough* OR plow* OR till* OR "direct drill*" OR fertili* OR biosolid* OR "bio solid" OR organic OR manur* OR sewage OR compost* OR amendment* OR biochar* OR digestate* OR "crop residue*" OR "crop straw*" OR mulch* OR "crop rotat*" OR "break crop*" OR "grass ley" OR "clover ley" OR legume* OR "bioenergy crop*" OR "cover crop*" OR "grass clover" OR "cropping system*" OR "crop system" OR "winter crop*" OR "spring crop*" OR "summer fallow*" OR "catch crop*" OR intercrop* OR conservation) AND (CH4 OR methane OR CO2 OR "carbon dioxide" OR N2O OR "nitrous oxide" OR GHG* OR "greenhouse gas*" OR "green-house gas*") AND (flux* OR dynamic* OR emission* OR exchang* OR balanc*)) | - 1900-2019 - Web of Science Core Collection - Topic field - All languages - All document types - Exclude: Oceanography (125), Nanoscience (190), Marine Freshwater biology (156), Economics (112), Computer science (87) - Institution subscriptions (Carleton University): - Science Citation Index Expanded (1900 - present) - Social Sciences Citation Index (1956 - present) - Arts & Humanities Citation Index (1975 - present) - Conference Proceedings Citation Index - Science (1990 - present) - Conference Proceedings Citation Index - Social Science and Humanities (1990 - present) | 16,993  [Mar 12, 2019] |

### PubMed

Note: Command line advanced search selected to search within: title, abstract, keywords.

Table 2. Metadata from PubMed Search Strategy

| Search string | Restrictions | Returns  [Date] |
| --- | --- | --- |
| (((arable OR agricult* OR farm OR farms OR farming OR crop* OR cultivat* OR field*)) AND (plough* OR plow* OR till OR tills OR tillage OR tilling OR tilled OR "direct drill*" OR fertili* OR biosolid* OR "bio solid" OR organic OR manur* OR sewage OR compost* OR amendment* OR biochar* OR digestate* OR "crop residue*" OR "crop straw*" OR mulch* OR "crop rotat*" OR "break crop*" OR "grass ley" OR "clover ley" OR legume* OR "bioenergy crop*" OR "cover crop*" OR "grass clover" OR "cropping system*" OR "crop system" OR "winter crop*" OR "spring crop*" OR "summer fallow*" OR "catch crop*" OR intercrop* OR conservation)) AND (CH4 OR methane OR CO2 OR "carbon dioxide" OR N2O OR "nitrous oxide" OR GHG* OR "greenhouse gas*" OR "green-house gas*")) AND (((flux* OR dynamic* OR emission* OR exchang* OR balanc*))) | - All years - All languages - Performed search in EPPI using PubMed Import tool - Institutional Subscription (Carleton University): over 11 million citations in MEDLINE, PreMEDLINE and other related databases, with links to participating online journals. | 3,446  [Mar 14, 2019] |

### Scopus

Note: Advanced search selected to search within: title, abstract, and keywords.

Table 3. Metadata from Scopus Search Strategy

| Search string | Time restrictions | Returns  [Date] |
| --- | --- | --- |
| TITLE-ABS-KEY((arable OR agricult* OR farm* OR crop* OR cultivat* OR field*) AND (plough* OR plow* OR till* OR "direct drill*" OR fertili* OR biosolid* OR "bio solid" OR organic OR manur* OR sewage OR compost* OR amendment* OR biochar* OR digestate* OR "crop residue*" OR "crop straw*" OR mulch* OR "crop rotat*" OR "break crop*" OR "grass ley" OR "clover ley" OR legume* OR "bioenergy crop*" OR "cover crop*" OR "grass clover" OR "cropping system*" OR "crop system" OR "winter crop*" OR "spring crop*" OR "summer fallow*" OR "catch crop*" OR intercrop* OR conservation) AND (CH4 OR methane OR CO2 OR "carbon dioxide" OR N2O OR "nitrous oxide" OR GHG* OR "greenhouse gas*" OR "green-house gas*") AND (flux* OR dynamic* OR emission* OR exchang* OR balanc*)) | - All years (1823-2019) - English only - All document types - Exclude: Social Science (546), Medicine (394), Business (341), Computer Science (191), Economics (171), Pharmacology (134), Mathematics (111) - Institution Subscriptions (Carleton University) - Some article backfiles and their cited references from 1970 | 14,987  [Mar 12, 2019] |

### Directory of Open Access Journals

Table 4. Metadata from Directory of Open Access Journals Search Strategy

| Search string | Time restrictions | Returns  [Date] |
| --- | --- | --- |
| Arable AND (greenhouse gas)  Agriculture AND (greenhouse gas) | - All years (1990-2018) - All Categories - Full record searches - No Institutional subscription required | 38 [May 23, 2019]  797 [May 23, 2019] |

### ProQuest Dissertations and Theses Global

Table 5. Metadata from ProQuest Dissertations and Theses Global Search Strategy

| Search string | Time restrictions | Returns  [Date] |
| --- | --- | --- |
| TI,AB,IF((arable OR agricult* OR farm* OR crop* OR cultivat* OR field*) AND (plough* OR plow* OR till* OR "direct drill*" OR fertili* OR biosolid* OR "bio solid" OR organic OR manur* OR sewage OR compost* OR amendment* OR biochar* OR digestate* OR "crop residue*" OR "crop straw*" OR mulch* OR "crop rotat*" OR "break crop*" OR "grass ley" OR "clover ley" OR legume* OR "bioenergy crop*" OR "cover crop*" OR "grass clover" OR "cropping system*" OR "crop system" OR "winter crop*" OR "spring crop*" OR "summer fallow*" OR "catch crop*" OR intercrop* OR conservation) AND (CH4 OR methane OR CO2 OR "carbon dioxide" OR N2O OR "nitrous oxide" OR GHG* OR "greenhouse gas*" OR "green-house gas*") AND (flux* OR dynamic* OR emission* OR exchang* OR balanc*)) | - All years (1861-2019) - Dissertations & Theses Global - Master’s and doctoral dissertation - Institutional Subscription (Carleton University): - Indexing 1743 - present; Full text 1997 – present - Comprehensive collection of dissertations and theses from around the world, spanning from 1743 to the present. PQDT Global includes theses from Great Britain and Ireland. | 1,085  [March 15, 2019] |

### ETHOS

Table 6. Metadata from ETHOS Search Strategy

| Search string | Time restrictions | Returns  [Date] |
| --- | --- | --- |
| Agriculture AND Greenhouse gas  Arable AND Greenhouse gas | - All years (1800-2019) - Any word (everywhere but full-text) - Doctoral Theses only - No institutional subscription required | 68 [May 15, 2019]  15 [May 15, 2019] |

### AGRIS (FAO)

Table 7. Metadata from AGRIS Search Strategy

| Search string | Time restrictions | Returns  [Date] |
| --- | --- | --- |
| Agriculture AND Greenhouse gas  Arable AND Greenhouse gas | - All years - Publications and Datasets - No institutional subscription required | 799 [May 24, 2019]  81 [May 24, 2019] |

### Google Scholar

Table 8. Metadata from Google Scholar Search Strategy

| Search string | Time restrictions | Returns  [Date] |
| --- | --- | --- |
| arable "greenhouse gas" OR methane OR "carbon dioxide" OR "nitrous oxide" OR CH4 OR CO2 OR N2O  agriculture "greenhouse gas" OR methane OR "carbon dioxide" OR "nitrous oxide" OR CH4 OR CO2 OR N2O | - All years - No institutional subscription required | 250 [July 12, 2019]  250 [July, 12, 2019] |

### Website Searches

The first 50 results, sorted by relevance, from each of the websites below using the following simplified search strings were screened.

| **Organization** | **Website URL** | **Search Date** | **Search String Used** |
| --- | --- | --- | --- |
| Department of Agriculture, Environment, and Rural Affairs (Northern Ireland) | [https://www.daera-ni.gov.uk](https://www.daera-ni.gov.uk/) | March 9th 2019 | arable AND greenhouse gas |
|  |  |  | arable AND CO2 |
|  |  |  | arable AND CH4 |
|  |  |  | arable AND NO2 |
|  |  |  | agriculture AND greenhouse gas |
|  |  |  | agriculture AND CO2 |
|  |  |  | agriculture AND CH4 |
|  |  |  | agriculture AND NO2 |
| Environmental Protection Agency (Ireland) | <http://epa.ie/> | March 10th 2019 | arable (AND) greenhouse gas |
|  |  |  | agriculture (AND) greenhouse gas |
| Centre for Ecology & Hydrology (UK) | <https://www.ceh.ac.uk/> | March 10th 2019 | arable (AND) greenhouse gas |
|  |  |  | agriculture (AND) greenhouse gas |
| European Environment Agency | <https://www.eea.europa.eu/> | March 10th 2019 | arable AND greenhouse gas |
|  |  |  | agriculture AND greenhouse gas |
| EU Science Hub | <https://ec.europa.eu/jrc/en> | March 10th 2019 | agriculture AND greenhouse gas |
|  |  |  | arable AND greenhouse gas |
| National Trust (UK) | <https://www.nationaltrust.org.uk/> | March 10th 2019 | arable (AND) greenhouse gas |
|  |  |  | agriculture (AND) greenhouse gas |
| British Society of Soil Sciences (UK) | <https://soils.org.uk/> | March 11th 2019 | arable AND greenhouse gas |
|  |  |  | arable AND CO2 |
|  |  |  | arable AND CH4 |
|  |  |  | arable AND N2O |
|  |  |  | agriculture AND greenhouse gas |
|  |  |  | agriculture AND CO2 |
|  |  |  | agriculture AND CH4 |
|  |  |  | agriculture AND N2O |
| Sniffer (Scotland) | <https://www.sniffer.org.uk> | March 12th 2019 | arable AND greenhouse gas |
|  |  |  | arable AND CO2 |
|  |  |  | arable AND CH4 |
|  |  |  | arable AND N2O |
|  |  |  | agriculture AND greenhouse gas |
|  |  |  | agriculture AND CO2 |
|  |  |  | agriculture AND CH4 |
|  |  |  | agriculture AND N2O |
| Project Drawdown | <https://www.drawdown.org/> | March 12th 2019 | agriculture |
| Scottish Environment Protection Agency | <https://www.sepa.org.uk/> | March 12th 2019 | arable AND greenhouse gas |
|  |  |  | arable AND CO2 |
|  |  |  | arable AND CH4 |
|  |  |  | arable AND N2O |
|  |  |  | agriculture AND greenhouse gas |
|  |  |  | agriculture AND CO2 |
|  |  |  | agriculture AND CH4 |
|  |  |  | agriculture AND N2O |
| Scottish Government | <https://www.gov.scot/> | March 12th 2019 | agriculture AND greenhouse gas |
|  |  |  | agriculture AND CO2 |
|  |  |  | agriculture AND CH4 |
|  |  |  | agriculture AND N2O |
|  |  |  | arable AND greenhouse gas |
|  |  |  | arable AND CO2 |
|  |  |  | arable AND CH4 |
|  |  |  | arable AND N2O |
| United Kingdom Government | <https://www.gov.uk/search/> | March 15^th^, 2019 | agriculture AND greenhouse gas |
|  |  |  | arable AND greenhouse gas |
| Rothamsted Research | <https://repository.rothamsted.ac.uk/repository> | March 15^th^, 2019 | agriculture AND greenhouse gas |
|  |  |  | arable AND greenhouse gas |
| Natural Resources Wales | [https://naturalresources.wales](https://naturalresources.wales/) | March 15^th^, 2019 | agriculture AND greenhouse gas |
|  |  |  | arable AND greenhouse gas |
